# Recurrence in skeletal muscle from squamous cell carcinoma of the uterine cervix: a case report and review of the literature

**DOI:** 10.1186/1471-2407-6-169

**Published:** 2006-06-27

**Authors:** Gabriella Ferrandina, Vanda Salutari, Antonia Testa, Gian Franco Zannoni, Marco Petrillo, Giovanni Scambia

**Affiliations:** 1 Gynecologic Oncology Unit, Catholic University, L.go Gemelli 8, 00168, Rome, Italy; 2 Institute of Human Pathology, Catholic University, L.go Gemelli 8, 00168, Rome, Italy; 3 Department of Oncology, Catholic University, Contrada Tappino, Campobasso, Italy

## Abstract

**Background:**

The occurrence of skeletal muscle metastases is a very rare event. Only two cases of late skeletal muscle recurrence from cervical cancer have been documented until now.

**Case presentation:**

A 38-year old patient, submitted to radical hysterectomy and pelvic lymphadenectomy for a squamous FIGO stage IB1 cervical carcinoma, presented after 76 months with a palpable, and painless swelling on the left hemithorax. MRI showed a nodule located in the context of the intercostal muscles. Pathology revealed the presence of metastasis of squamous cell carcinoma of similar morphology as the primary. On the basis of FDG-PET findings, which excluded other sites of disease, surgical excision of the lesion was performed. The patient was triaged to chemotherapy plus external radiotherapy.

**Conclusion:**

A case of skeletal muscle recurrence from cervical cancer after a very long interval from primary diagnosis is reported. Muscular pain or weakness, or just a palpable mass in a patient with a history of cancer has always to raise the suspicion of muscle metastasis.

## Background

The incidence of skeletal muscle metastases is reported to be less than 1% of metastases of haematogenous origin, despite the fact that skeletal muscle accounts for nearly 50% of the total body weight and is characterized by a rich blood supply [1]. The reasons for the rarity of metastatic involvement of skeletal muscle are still unclear, but several hypotheses have been made including: a) the constant movement of skeletal muscles, which may represent a difficult condition for the implantation and growth of metastatic cells under the high tissue pressure related to the exercise associated increase of blood flow; b) the local production of lactic acid, which would create an unfavourable environment for metastatic cell growth; c) the inhibition of cell invasion by protease inhibitors located in the basement membrane; d) the antitumor activity of lymphocytes and/or natural killer cells within the skeletal muscle; e) in vivo evidences that skeletal muscle-delivered peptidic factors may influence the metastatization process [1]. Psoas, iliopsoas and paraspinal muscles, and proximal musculature of the upper and lower limbs, represent the most frequently involved sites. Malignancies known to frequently metastasize to the muscle are melanoma, kidney, lung, thyroid cancer; moreover skeletal muscle metastases have been also reported in lymphoma, leukaemia and colon cancer patients [1].

To our knowledge, five cases of skeletal muscle metastasis and two cases of skeletal muscle recurrence after 24 and 60 months fro initial diagnosis of cervical cancer have been documented until now [2-9].

We report an unusual case of isolated intercostal muscle recurrence after a very long interval from primary diagnosis of squamous cervical cancer. A review of the literature with particular emphasis on the diagnostic and therapeutic issues is also presented.

## Case presentation

A 38-year old patient was admitted to the Gynaecologic Oncology Unit of the Catholic University of Rome in June 1998 for irregular vaginal bleeding. Gynaecological examination revealed an ulcerated lesion (maximum diameter = 3 cm) of the uterine cervix The histopathologic diagnosis of colposcopy-guided biopsy revealed the presence of moderately differentiated squamous cell cervical carcinoma. The patient was clinically staged as having FIGO stage IB1 cervical cancer and was submitted to radical hysterectomy and pelvic lymphadenectomy. Final staging was pT1bN0M0 according to TNM classification.

**Figure 1:**
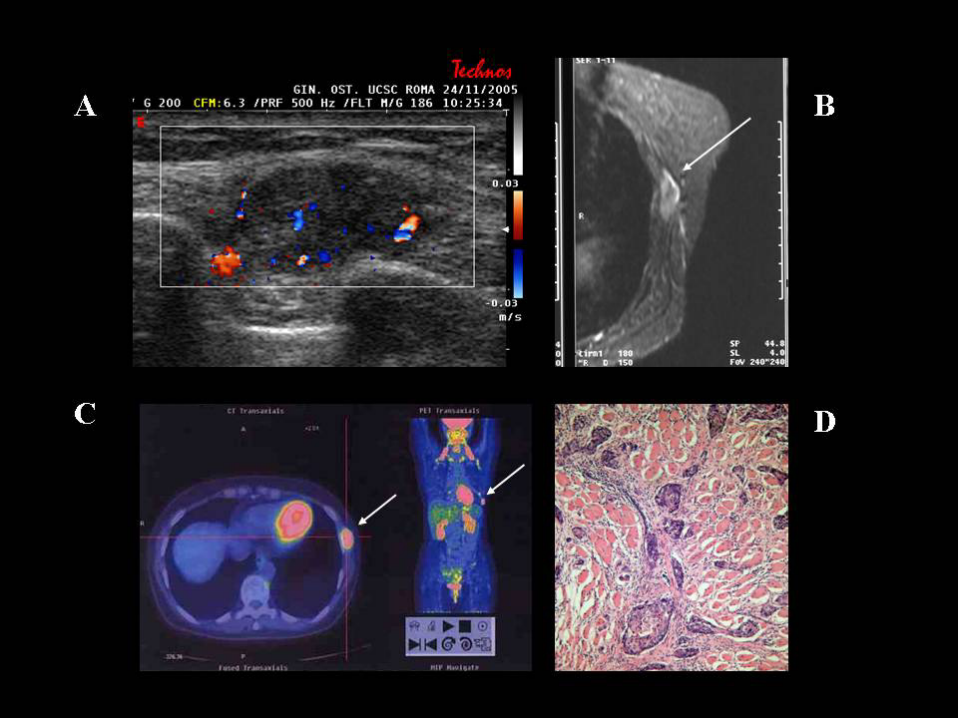
**A**. Ultrasonography of the left thorax documenting a hypoechogenic nodule in the context of the intercostal muscle, close to the underlying rib. The nodule shows intralesional vessels at Color Doppler examination. **B**. T1-weighted MRI showing the presence of a moderate gadolinium-contrast enhancement area in the intercostal muscle adjacent to the rib. **C**. FDG-PET/CT scan documenting two focuses of abnormal FDG uptake in the left hemithorax. **D**. Recurrence of squamous cell cervical carcinoma in the rib cage muscle. The bands of skeletal muscle are separated and infiltrated by nests of squamous carcinoma cells surrounded by desmoplasic stroma and showing areas of keratinisation. (Hematoxylin & Eosin, magnification 100×).

After 76 months from initial diagnosis, the patient presented with a palpable, painless, mobile and tender swelling on the left side of the thorax approximately at the level of the 5th rib. Ultrasonography was performed revealing a subfascial hypoechogenic lesion (maximum diameter = 2 cm) within the intercostal muscles, close to the rib, which however, appeared uninvolved (Figure 1A). Magnetic Resonance Imaging (MRI) study of the left rib cage confirmed the presence of an intermediate intensity signal area in both T1 and T2-weighted images, with a moderate gadolinium-contrast enhancement, which was considered consistent with a desmoid lesion (Figure 1B). Gynecological examination as well as chest X-ray and MRI of the abdomen and pelvis were normal, and circulating levels of SCC were negative (0.8 ng/ml). Fine-needle aspiration of the lesion was planned and revealed the presence of squamous carcinoma cells. The patient was then investigated by fluorodeoxyglucose positron emission tomography (FDG-PET), which showed only two abnormal uptakes of the radiotracer in the left hemithorax (Figure 1C). On the basis of FDG-PET findings, which excluded other sites of metastatic disease, a wide surgical excision of the lesion was performed and final pathology revealed the presence of a nodule (maximum diameter = 1.7 cm) consistent with metastasis of squamous cell carcinoma (Figure 1D) of similar morphology as the primary. The surgical margins were free of disease.

After extensive counselling, the patient was triaged to combined chemotherapy with carboplatin (5AUC) and paclitaxel (175 mg/m2), plus external radiotherapy (total dose = 4,000 cGy in 20 fractions) on the left hemi thorax. After two months from the completion of treatment, the patient shows no evidence of disease and does not complain of any symptom related to radiation treatment.

## Conclusion

We first reported a case of isolated intercostal cage muscle recurrence after a very long interval from the diagnosis of squamous cell carcinoma of the uterine cervix. As shown in Table 1, skeletal muscle involvement from cervical carcinoma is very rare, and usually documented in the context of an already far advanced stage tumor or metastatic disease [6-8], or in severely immunocompromised patients, as occurs in patients with AIDS [3,4]. In addition, an unusual case of involvement of masseter muscle has been also described in a case with very aggressive histology [5]. Although the association between specific tumor histotypes and tendency to involve skeletal muscle has been not determined, in lung cancer skeletal muscle metastasis have been documented to more frequently develop from adenosquamous cell carcinoma [10]. The clinical outcome of patients with skeletal muscle metastasis is generally poor [1,10], given the common finding of diffuse metastatic disease. As shown in Table 1, most of the data about the clinical course of cervical cancer patients with skeletal muscle involvement is lacking.

Establishing the diagnosis of skeletal muscle metastasis sometimes can be difficult: indeed, although skeletal muscle metastases can be painful or palpable or cause deformity according to location, sometimes they can be asymptomatic. Indeed, our patient only complained of a mobile, painless swelling, which could have been easily underestimated given the long interval from initial diagnosis, the favourable clinical pathological characteristics of disease (squamous histotype, FIGO stage IB1), and, above all, the unusual and unexpected recurrence presentation. Although the histopathological evaluation represents a more straight forward diagnostic approach, diagnostic imaging techniques are useful tools in order to define the site and extension of disease [11], and exclude diffuse metastases. However, the differential diagnosis between primary intramuscular tumor and metastasis, which is a very important issue for patient prognosis and clinical management, is not easy: in our case MRI showed an intermediate signal area intensity on T1 and T2-weighted scans, very similar to the muscle tissue but with an important enhancement after the contrast injection. None of the diagnostic tools used documented any signs of the presence of necrosis or haemorrhage. which would have been suggestive of a pattern of rapid growth. In this context, much attention has been focused on the usefulness of FDG-PET not only for staging, but also for the detection of recurrence in cervical carcinoma: in our case FDG-PET findings showed to be critical in order to plan patient management since a diffuse metastatic disease was excluded, and surgical removal of the intercostal muscle recurrence could be planned. Given the very rare occurrence of skeletal muscle metastasis, there are no specific guidelines on the therapeutic options, which include radiotherapy, chemotherapy and surgery according to the clinical setting: indeed, the real benefit of these approaches remains questionable given the very poor prognosis of these patients. As shown in Table 1, in case of a solitary and surgically resectable skeletal muscle metastasis, metastasectomy has been performed, followed by radiotherapy [9]. In our case the choice of a multimodal approach was sustained by i)the young age of the patient and her good performance status; i) the documentation of a single and surgically amenable recurrence; ii) the general consideration of skeletal muscle metastasis as a marker of systemic disease, which would require chemotherapy: in particular the platinum-taxane combination was chosen on the basis of the high response rate documented with this regimen with respect to cisplatin alone [12]; the choice of using carboplatin instead of cisplatin was motivated by the documentation of a better patient convenience and tolerance together with the encouraging results obtained with carboplatin/paclitaxel regimen in advanced/recurrent pre-irradiated cervical cancer patients [13]. In addition, radiotherapy was planned in order to achieve a better local control of disease, after balancing and discussing with the patient the overall risk of pulmonary fibrosis related to the specific site of irradiation.

In conclusion, we reported a case of a solitary distant skeletal muscle recurrence from early stage cervical cancer after a very long interval from primary diagnosis. Late recurrence (i.e. after a disease-free interval of more than 5 years) in cervical cancer patients is considered a very rare event occurring in approximately 4% of cases with a frequency increasing from 1.8% in stage I, to 5.2% and 8.5% in stage II and III disease, respectively [14]. These figures suggest that cervical cancer patients, especially those bearing stage II-III disease warrant long follow up care, because of the long period of tumor cell dormancy. In particular, muscular pain or weakness, or just a palpable mass in a patient with a history of cervical cancer has always to raise the suspicion of metastatic muscular disease.

## Competing interests

The author(s) declare that they have no competing interests.

## Authors' contributions

GF conceived the study, was the coordinator of the study, and drafted the manuscript

VS collected the clinical data

AT was responsible for the clinical surveillance

GFZ was responsible for the evaluation of histopathological data

MP participated in the coordination of the study, and in drafting the manuscript

GS participated in the draft of the study, and in the conception of the study

All authors read and approved the final manuscript

## Pre-publication history

The pre-publication history for this paper can be accessed here:



## Figures and Tables

**Table 1:** Characteristics of cervical cancer patients with skeletal muscle involvement, as reported in the literature

**Author**	**Primary tumor histotype**	**Stage**	**Interval from primary tumor (months)**	**Skeletal muscle**	**HIV status**	**Treatment**	**Clinical outcome (after diagnosis of metastasis)**
Mariya [2]	squamous	n.a.	n.a.	Biceps	negative	n.a.	n.a.
Schwartz [3]	squamous	IIIB	0	Iliopsoas	positive	Chemotherapy followed by radiotherapy	DOD after 3 months
Singh [4]	squamous	IIIB	0	Psoas	positive	Patient declined	n.a.
Wong [5]	small cell	IB	24	Masseter	negative	Chemotherapy + radiotherapy	n.a.
Bar Dayan [6]	squamous	IIIB	0	Psoas	negative	Intralesional chemotherapy followed by radiotherapy	DOD after 4 months
Pathy [7]	squamous	IIIB	0	Deltoid	negative	Palliative radiotherapy	ED at time of publication
Devendra [8]	squamous	n.a.	0	Psoas	negative	Supportive therapy	Lost to follow up
Saadi [9]	squamous	IIB	60	Psoas	negative	Metastasectomy, radiotherapy	NED after 30 months
Current case	squamous	IB	76	Intercostal	negative	Metastasectomy followed by chemotherapy + radiotherapy	NED after 8 months

n.a. = not available; DOD = death of disease; ED = alive with evidence of disease; NED = no evidence of disease
